# Understanding the Effects of Ultrasound (408 kHz) on the Hydrogen Evolution Reaction (HER) and the Oxygen Evolution Reaction (OER) on Raney-Ni in Alkaline Media

**DOI:** 10.1016/j.ultsonch.2022.105979

**Published:** 2022-03-11

**Authors:** Faranak Foroughi, Christian Immanuel Bernäcker, Lars Röntzsch, Bruno G. Pollet

**Affiliations:** a Hydrogen Energy and Sonochemistry Research Group, Department of Energy and Process Engineering, Faculty of Engineering, Norwegian University of Science and Technology (NTNU), Trondheim NO-7491, Norway; b Fraunhofer Institute for Manufacturing Technology and Advanced Materials IFAM, Branch Lab Dresden, Winterbergstraße 28, Dresden 01277, Germany; c Green Hydrogen Lab (GH2Lab), Pollet Research Group, Hydrogen Research Institute, Université du Québec à Trois-Rivières, 3351 Boulevard des Forges, Trois-Rivières, Québec G9A 5H7, Canada

**Keywords:** Hydrogen evolution reaction (HER), Oxygen evolution reaction (OER), Raney-Nickel, Alkaline, Ultrasound, Sonoelectrochemistry

## Abstract

The hydrogen evolution reaction (HER) and the oxygen evolution reaction (OER) occurring at the Raney-Ni mesh electrode in 30 wt.-% aqueous KOH solution were studied in the absence (*silent*) and presence of ultrasound (408 kHz, ∼54 W, 100% acoustic amplitude) at different electrolyte temperatures (*T* = 25, 40 and 60 °C). Linear sweep voltammetry (LSV) and electrochemical impedance spectroscopy (EIS) experiments were performed to analyse the electrochemical behaviour of the Raney-Ni electrode under these conditions. Under *silent* conditions, it was found that the electrocatalytic activity of Raney-Ni towards the HER and the OER depends upon the electrolyte temperature, and higher current densities at lower overpotentials were achieved at elevated temperatures. It was also observed that the HER activity of Raney-Ni under ultrasonic conditions increased at low temperatures (e.g., 25 °C) while the ultrasonic effect on the OER was found to be insignificant. In addition, it was observed that the ultrasonic effect on both the HER and OER decreases by elevating the temperature. In our conditions, it is suggested that ultrasound enhances the electrocatalytic performance of Raney-Ni towards the HER due to principally the efficient gas bubble removal from the electrode surface and the dispersion of gas bubbles into the electrolyte, and this effect depends upon the behaviour of the hydrogen and oxygen gas bubbles in alkaline media.

## Highlights

• Under *silent* conditions, the HER and OER activities of Raney-Ni in weak alkaline media improve when the electrolyte temperature is increased.
• The HER activity of Raney-Ni in weak alkaline media under ultrasonic conditions (408 kHz) increases at 25 °C while the OER activity of Raney-Ni under ultrasonic conditions is not affected.
• Combining ultrasonication (408 kHz) at high electrolyte temperatures do not improve the HER and OER activities of Raney-Ni in weak alkaline media.
• Ultrasound (408 kHz) has no influence on the stability of the Raney-Ni coating.

## 1 Introduction

Water electrolysis is the most significant primary electrochemical method for molecular hydrogen (H_2_) production, and its importance is increasing rapidly with affordable renewable energy production [1]. The electrolysis of water involves two half-cell reactions: the hydrogen evolution reaction (HER) taking place at the cathode (the negative electrode) and the oxygen evolution reaction (OER) at the anode (the positive electrode) [2]. Depending on the electrolytes, separators, working temperatures and pressures employed, currently, there are five main types of water electrolysers, namely [1], [2]:

1. Proton exchange membrane water electrolyser (PEMWE, liquid water, perfluorosulfonic acid (PFSA), < 80 °C, < 200 bar);
2. Alkaline water electrolyser (AWE, 30-40% KOH or NaOH, < 80 °C, < 30 bar);
3. Anion exchange membrane water electrolyser (AEMWE, dilute KOH, < 90 °C, < 30 bar);
4. Solid oxide electrolysis cell (SOEC, water steam, 500-850 °C, atmospheric); and,
5. Proton conducting ceramic electrolyser (PCCEL, water stream, 300-600 °C, < 8 bar).

PEMWEs are still expensive ($1,000 – $2,000/kW) [1], [2] due to the high cost of precious metals (mainly iridium, Ir and platinum, Pt) and other materials such as the polymeric proton exchange membrane (e.g., Nafion®). AWE is a proven technology offering advantages such as the use of inexpensive metals (e.g., nickel, Ni) and materials and lower manufacturing costs and operations [1], [2]. It is expected that the cost of AWE will drop significantly in the next 5 years by optimizing stack design and developing more efficient and long-term stable electrodes, made from inexpensive raw materials, and produced by mass fabrication-suitable processes [1], [2], [3].

Raney-type electrodes are made of Ni-Zn and Ni-Al precursor alloys, producing high surface area after leaching in alkaline solutions [4], [5]. Raney-Ni electrodes have shown well-proven good electrocatalytic activity towards the HER (Eq. (1)) and the OER (Eq. (2)) in alkaline electrolytes [6], [7], [8].

(1) $$\mathrm{Cathodic}\;reaction:4H_{2}O\left(l\right)+4e^{-}\to 2H_{2}\left(g\right)+4OH^{-}\left(aq\right)$$

(2) $$\mathrm{Anodic}\;reaction:4OH^{-}\left(aq\right)\to O_{2}\left(g\right)+2H_{2}O\left(l\right)+4e^{-}$$

In water electrolysis, the cell voltage (*V*_cell_) is a crucial factor representing energy consumption and is expressed in Equation (3), where *E*_a_ is the anode potential for the OER, *E*_c_ is cathode potential for the HER, *j* is the current density, ∑R is total ohmic resistance, *E*^rev^ is the theoretical reversible potential (*Nernst*), *η*_a_ is the anode overpotential, and *η*_c_ is the cathode overpotential [9].

(3) $$V_{\mathrm{cell}}=\left|E_{c}-E_{a}\right|+j\times \sum R={\Delta E}^{\mathrm{rev}}+\left|\eta_{a}\right|+\left|\eta_{c}\right|+j\times \sum R$$

According to Equation (3), *V*_cell_ is comprised of three components, the theoretical reversible cell voltage (Δ*E*^rev^), the total cell overpotential (Σ*η*) and the Ohmic cell voltage drop (*j*Σ*R*)*.* For increasing the water electrolysis rate, reducing the total overpotential is essential to overcome the energy barrier, and thus electrode materials and the effective electrode surface area play a crucial role on reaction overpotential. Another important factor leading to high energy consumption in water electrolysis is the Ohmic voltage drop which is expressed in Equation (4).

(4) $$\sum R=R_{e}+R_{m}+R_{b}+R_{c}$$

where *R*_e_ is the electrolyte resistance, *R*_m_ is the membrane/separator resistance, *R*_b_ is the bubble resistance and *R_c_* is the external circuit resistance. The *R*_m_ and *R*_c_ are constant in water electrolysis, which can be reduced by optimizing cabling connection and membrane/separator production process. The dispersion of the bubbles in the electrolyte decreases the electroanalyte conductivity and in turns increases *R*_e_. In addition, the bubble coverage on the electrode surface act as an insulating layer, reducing the effective surface area of the electrode, yielding high bubble resistance *R*_b_. In most cases, the Ohmic voltage drop can be minimized by increasing the electrolyte flowrate, using gravity, using a magnetic field at the gas-evolving electrodes or by applying ultrasound [9].

Overall, the efficiency of water electrolysis can be improved by: (i) enabling the detachment of gas bubbles from the electrodes and the membranes more effective, thereby eliminating gas blanketing; (ii) by promoting faster removal of the bubbles at the electrodes to increase the local heat/mass transfer coefficients; and (iii) by allowing efficient electrolyte degassing, even with very small electrode spacing.

Ultrasound is an acoustic wave that has a frequency above the upper limit of the human hearing range. Power ultrasound or low-frequency ultrasound is a well-defined sound wave in the range of [20 kHz – 2 MHz] and it is regarded as the effects of the sound wave on the medium [9], [10], [11]. The most important phenomenon that arises from the propagation of an ultrasonic wave into a liquid is acoustic cavitation [12]. When an ultrasonic wave propagates through a liquid media such as water, many tiny gas bubbles are formed. The phenomenon of the formation of bubbles and their subsequent violent collapse of the bubbles is known as *acoustic cavitation* [12]*.* The collapsing bubble can generate high temperatures up to 5,000 °C and high pressures up to 2,000 atm [13]. The sonochemical process can occur in three regions as follows [14], [15]:

1. The interior of the cavitation bubble, alos called the gaseous region. Here, the cavitation of micro-sized bubbles generates free radicals (H^•^ and OH^•^) by water pyrolysis.
2. The region at the interface of the bubble and liquid (gas-liquid interface), where the generation of OH^•^ radicals is predominant.
3. The region of bulk liquid. In this case, the free radicals generated at the interface of the bubble/liquid region move to the bulk liquid, producing a secondary sonochemical reaction.

**Figure 1:**
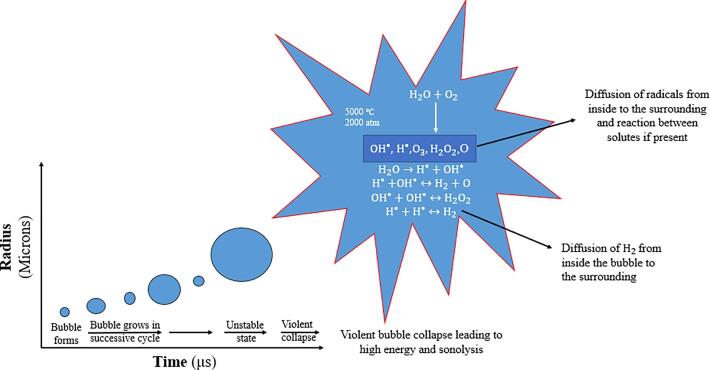
The evolution of a cavitation bubble during ultrasonication and the production of sonolysis species by acoustic cavitation.

The evolution of a cavitation bubble during ultrasonication is shown in Figure 1.

Sonoelectrochemistry is the combination of power ultrasound with electrochemistry. It has been shown that the use of ultrasound in electrochemistry offers many advantages including [10], [11]:

1. Gas bubble removal at the electrode surface.
2. Solution degassing.
3. Decreased adsorption process.
4. Increased chemical reaction rates.
5. Disruption of the *Nernst* diffusion layer.
6. Enhancement of mass transport of electroactive specious through the double layer.
7. Activation and cleaning of the electrode surface due to the erosion caused by cavitation bubble implosion.
8. Improvement of reaction mechanism by the production of free radicals through the cavitation process.

Water electrolysis under ultrasonication was first studied by Moriguchi in the 1930’s using a platinum (Pt) electrode and found that the process occurred at faster rates and lower cell voltages than under *silent* conditions [16]. Hydrogen production in the presence of ultrasound was then continued by the Pollet’s research group at the Birmingham Proton Exchange Membrane Fuel Cell in 2011 [10]. For example, Lepesant [17] and other researchers such as Zadeh [18] and Symes [19] studied the influence of ultrasonication on electrolytic hydrogen production from weak acidic (H_2_SO_4_) and alkaline (NaOH and KOH) solutions using various electrode materials including Pt, industrial carbon (C), glassy carbon (GC) and 316 stainless steel (316-SS). Pollet *et al.* [20] also investigated the effects of ultrasound (26 kHz) on the hydrogen evolution reaction (HER) in the mild acidic electrolyte on polycrystalline Pt. They showed that a 250% enhancement in current density at maximum acoustic power (∼30 W) through effective hydrogen bubble removal. Li *et al.* [21] studied the effects of power ultrasound on water electrolysis in various NaOH concentrations. They found that the energy efficiency of water electrolysis was considerably improved in the presence of an ultrasonic field. Overall, the energy-saving for molecular hydrogen production by using an ultrasonic field was found to be in the region of 10–25% for specific electrolyte concentrations, even when high current densities were employed.

Since water electrolysis is an important electrochemical process for generating hydrogen, the possibility of providing the basis for a more realistic cell design for water electrolysis in the presence of ultrasound is a valuable area of investigation [21]. In previous studies, in-depth kinetic analyses were not carried out to shed some light on the effects of ultrasound on the HER and the OER mechanisms as well as Tafel parameters in mild acidic and alkaline electrolytes. In this study, we have investigated the effects of ultrasound on the kinetics and mechanisms of HER and OER on Raney-Ni in 30 wt.-% aqueous KOH solution at different temperatures (*T* = 25 °C, 40 °C and 60 °C).

## 2 Experimental method

All electrochemical experiments were conducted using a potentiostat/galvanostat (BioLogic-SP 150) in a conventional three-electrode configuration using a 30 wt.-% KOH (Sigma-Aldrich, 99.99% in purity) solution at *T* = 25, 40 and 60 °C. All solutions were prepared by using ultra-high purity deionized water (Millipore, 18.2 MΩ cm in resistivity). Raney-Ni electrodes were synthesized by Fraunhofer IFAM and used as working electrodes (WE). The Raney-Ni electrodes were produced in three steps: a) spraying of an aqueous binder solution followed by the deposition of alumimim (Al) powder onto a Ni-mesh, b) heat-treatment to produce the Ni-Al phases (Ni_2_Al_3_ and NiAl_3_), and c) leaching of the electrodes. Their production and preparation are fully described elsewhere [7]. For each sonoelectrochemical experiment, a fresh Raney-Ni electrode was used. A Ni mesh (40 mesh woven from 0.13 mm diameter wire, 99.99% metal basis, Alfa Aesar, Germany) was cut out in a rectangle shape (20.67 × 10.76 mm) and used as a counter electrode (CE). The reference electrode (RE) was a mercury/mercury oxide (Hg/HgO) filled with 30 wt.% KOH solution (the same electrolyte). All potentials in this work are reported with respect to RHE (*E*_RHE_ = *E*_Hg/HgO_ + 0.90 V). Also, potential values were *IR* compensation corrected based upon Equation (5):

*E*_IR-corrected_ = *E* – *IR* (5)

where *I* is the measured current and *R* is the electrolyte resistance, measured for each electrolyte solutions employed. The *R* value was determined by electrochemical impedance spectroscopy (EIS) from the value of the *real* impedance (*Z^’^*) where the *imaginary* impedance (*Z*^’’^) is zero in the Nyquist plot. The EIS experiments were carried out from 100 kHz to 0.1 Hz with a voltage perturbation of ±10 mV at *T* = 25, 40 and 60 °C.

**Figure 2:**
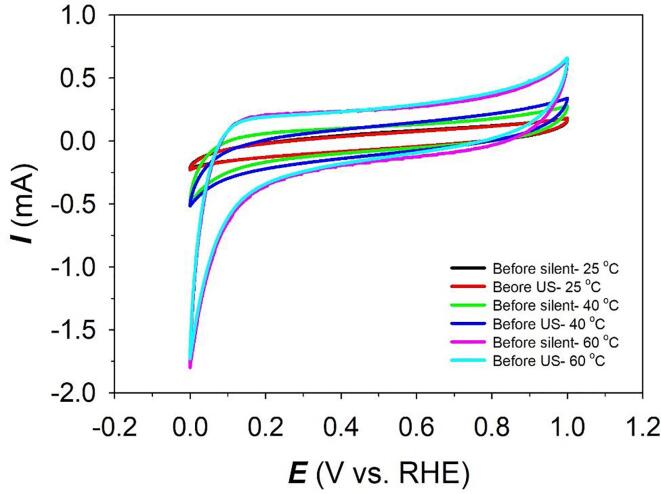
Pre-treatment CV profiles of Raney-Ni in 30 wt.-% aqueous KOH solution at a scan rate of *v* = 10 mV s^-1^ after purging with N_2_(g) for 30 min at *T* = 25, 40 and 60 °C.

All current densities are given in relation to the geometric surface area of the electrodes (*A*_geo_ = 0.085 cm^2^) and are referred to as *j*. The geometric surface area comprises only the front side of the electrode and neglecting the holes of the used mesh. Consequently, the surface area is underestimated [7]. The electrochemical surface area (*A*_ecsa_) was determined electrochemically by calculating the double-layer capacitance, *C*_dl_, from cyclic voltammetry (CV) experiments. The CV experiments were performed in the non-faradaic region at -0.55 to -0.45 V *vs.* Hg/HgO (+0.35 to +0.45 V *vs.* RHE). Before each experiment, a pre-treatment CV test was performed to remove all absorbed *H*-species from the electrode surface (Figure 2). An appropriate potential range of ±0.05 V and a series of scan rates from 1 to 0.02 V s^-1^ (in decreasing direction) were chosen. The average current densities, *j*_average_ (see Equation (6)), were plotted *versus* the scan rate, resulting in a straight line. The slope of the line corresponds to the double-layer capacitance, *C*_dl_. The electrochemical surface area was then calculated by using the specific capacitance density (*c*) of 40 μF cm^-2^ [27] and Equation (6) [26]:

(6) $$j_{\mathrm{average}}=\frac{j_{\mathrm{anodic}}-j_{\mathrm{cathodic}}}{2}$$

(7) $$A_{\mathrm{ecsa}}=\frac{C_{\mathrm{dl}}}{c}$$

After calculating the *A*_ecsa_ , the specific surface area (*A*_s_) was obtained. The *C*_dl_, *A*_ecsa_ and *A*_s_ of Raney-Ni electrodes at different temperatures are given in Table 1.

**Table 1:** Double-layer capacitance (*C*_dl_), electrochemical surface area (*A*_ecsa_) and specific surface area (*A*_s_) of Raney-Ni at various temperatures.

Temperature (°C)	*C*_dl_ (mC)	*A*_ecsa_ (cm^2^)	*A_s_* (cm^2^ g^-1^)
25	0.37	9.25	925
40	1.31	32.82	3,282
60	3.28	82.00	8,200

**Figure 3:**
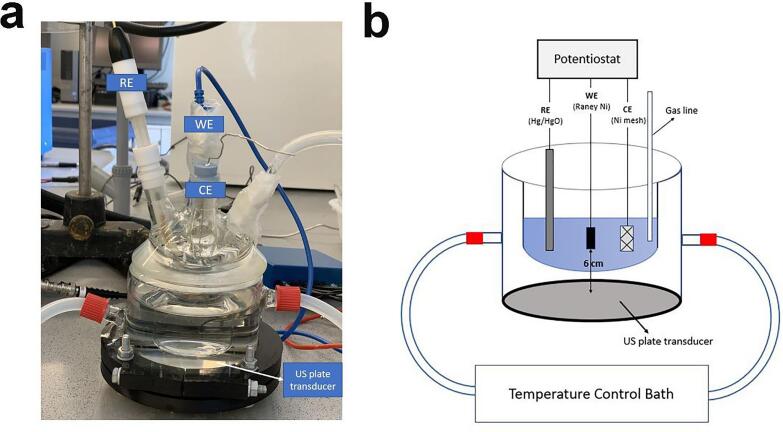
a) Experimental sonoelectrochemical set-up and b) schematic diagram of set-up.

The electrochemical studies were performed in a double-jacketed sonoelectrochemical cell (Figure 3) connected to a water bath (JULABO, GmbH) to keep the temperature at *T* = 25, 40 and 60 °C. The electrolyte was degassed with an ultra-high purity N_2_(g) (99.999% in purity) prior to and during the measurements. The WE was washed with ultra-high purity water before each sonoelectrochemical experiment. Ultrasonication was applied to the electrochemical cell by a plate transducer vibrating at a frequency *f* = 408 kHz (100% amplitude) powered by a multi-frequency ultrasonic generator (Meinhardt Ultrasonics). The ultrasonic or acoustic power (*P*_acoustic_) was determined calorimetrically using the methods of Margulis *et al.* [22] and Contamine *et al*. [23] and was found to be 54 ± 1.7 W.

Linear sweep voltammograms (LSV) were recorded at the potential region -0.80 ≤ *E*_app_ ≤ -1.50 V *vs.* Hg/HgO (+0.10 ≤ *E*_app_ ≤ -0.60 V *vs.* RHE) for the HER and +0.20 ≤ *E*_app_ ≤ +0.80 V *vs.* Hg/HgO (+1.10 ≤ *E*_app_ ≤ +1.70 V *vs.* RHE) for the OER experiments. The overpotentials for the OER at different temperatures were calculated based upon Equation (8):

(8) $$\eta =E_{\mathrm{app}}-E_{H2O}^{\mathrm{rev}}$$

where *E_app_* is the applied potential *vs.* RHE and $E_{H2O}^{\mathrm{rev}}$ corresponds to the theoretical (reversible) cell potential for decomposition of H_2_O at different temperatures. The temperature effect on the HER and OER has been discussed by many groups [24], [25], [26], [27], [28], [29]. A common understanding is based upon Equation (9) [29]:

(9) $$k=Aexp(\frac{-E_{a}}{\mathrm{RT}})$$

where *k* is the chemical reaction rate; *A* is the *Arrhenius* pre-exponential factor; *R* is the universal gas constant (8.314 J mol^-1^ K^-1^); *T* is the absolute temperature (K), and *E_a_* is the apparent activation energy (J mol^-1^). Equation (9) suggests that the higher the temperature, the faster the reaction rate so that larger current densities (at the same overpotential (*η*)) can be achieved at higher temperatures [29].

For the inspection of the electrodes’ cross-section, scanning electron microscopy (SEM) measurements were performed using a Jeol JSM F100 equipped with a field emission gun coupled to a Bruker Quantax 200 EDS spectrometer.

## 3 Results and discussion

### 3.1 Scanning electron microscopy (SEM) characterization of Raney-Ni before and after ultrasonication

**Figure 4:**
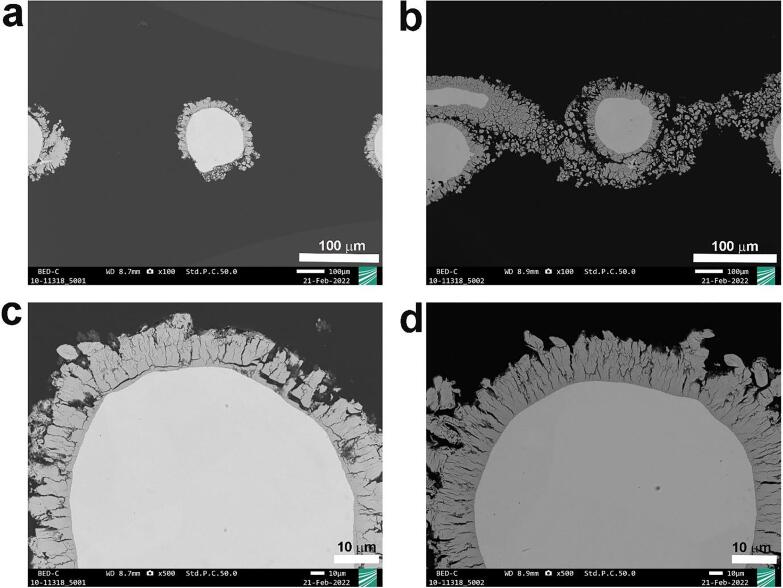
Cross-section SEM images of Raney-Ni at different magnifications: a) and c) without ultrasonication (*silent* conditions), b) and d) after ultrasonication for 15 min in 30 wt.-% aqueous KOH solution at *T* = 25 °C.

**Figure 5:**
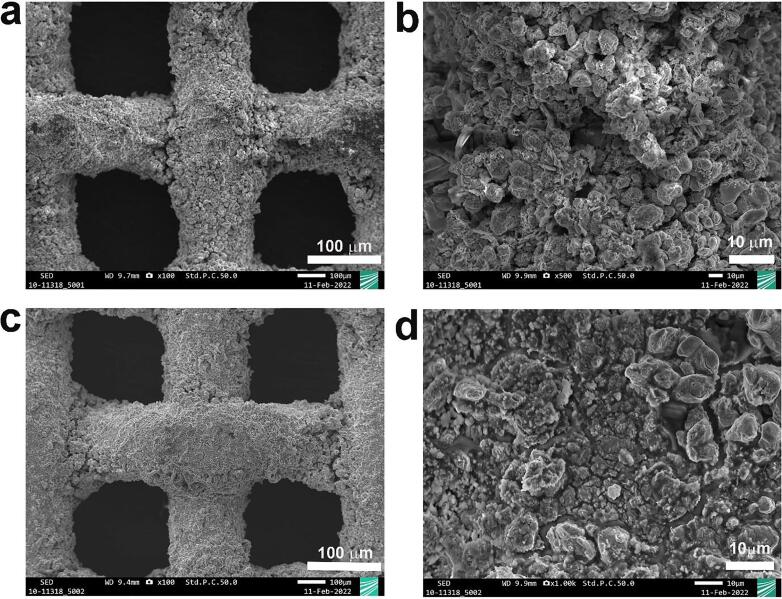
Top-view SEM images (SE mode) of Raney-Ni at different magnifications: a) and b) without ultrasonication (*silent* conditions), c) and d) after ultrasonication for 15 min in 30 wt.-% aqueous KOH solution *T* = 25 °C.

In order to see the stability of the coating layer of Raney-Ni under ultrasonication, SEM measurements were performed. Figures 4a and 4c show the cross-sections of Raney-Ni after immersion in 30 wt.-% aqueous KOH solution for 15 min without ultrasound (*silent*) and Figure 4b and 4d represents the cross-section of Raney-Ni after 15 min ultrasonication in 30 wt.-% aqueous KOH solution at *T* = 25 °C. Figure 4a appears to be different to the other ones. However, this could be related to the position of the cut during cross-sectional preparation. It can be seen in Figure 4 that ultrasound does not influence the stability of the Raney-Ni coating and the cross-section shows no delamination of the Raney-Ni under ultrasonic conditions. Figure 5 underpins the information from the cross-section. Figures 5a and 5b illustrate top-view SEM images of Raney-Ni after immersion in 30 wt.-% aqueous KOH solution for 15 min in the absence of ultrasound and Figures 5c and 5d demonstrate SEM micrographs of Raney-Ni after 15 min ultrasonication in 30 wt.-% aqueous KOH solution at *T* = 25 °C. According to Figure 5, the Raney-Ni layer appears to be strongly connected to the substrate, confirming the stability of the Raney-Ni coating after ultrasonication.

### 3.2 Effect of power ultrasound and temperature on the hydrogen evolution reaction

**Figure 6:**
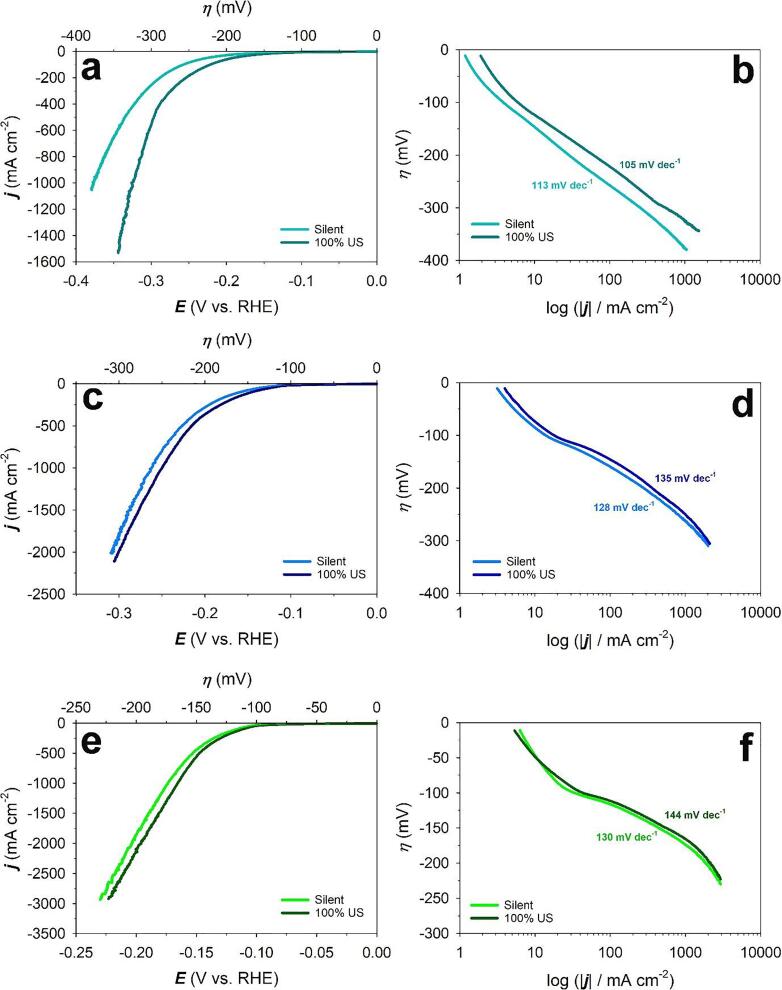
Linear sweep voltammograms (LSV) at a) 25 °C, c) 40 °C, e) 60 °C and Tafel plots of HER at b) 25 °C, d) 40 °C, f) 60 °C on Raney-Ni in 30 wt.-% aqueous KOH solution at a scan rate of *v* = 0.3 mV s^-1^ after purging with N_2_(g) for 30 min under *silent* and ultrasonic (US) conditions (*f* = 408 kHz).

To study the effect of power ultrasound on the hydrogen evolution reaction on Raney-Ni, linear sweep voltammetry (LSV) experiments were performed in the applied potential region of -0.80 ≤ *E*_app_ ≤ -1.50 V *vs.* Hg/HgO (+0.1 ≤ *E*_app_ ≤ -0.60 V *vs.* RHE) and at a very low potential scan rate *ν* = 0.30 mV s^-1^ to obtain a “quasi-steady-state” condition. Figure 6 shows the LSVs and the corresponding Tafel plots of Raney-Ni in the absence and presence of ultrasound at *T* = 25, 40 and 60 °C. In our conditions, it can be seen from the LSV curves (Figure 6-a,c and e) that the HER in the presence of ultrasound starts earlier for all temperatures used. Tafel slopes (*b*), exchange current densities (*j*_o_) and overpotential (*ƞ*) at -300 mA cm^-2^ obtained from the Tafel plots at different temperatures under *silent* and ultrasonic conditions are shown in Table 2. Table 2 shows that ultrasonication decreases the overpotential at -300 mA cm^-2^ by 34 mV (at 25 °C), 13 mV (at 40 °C) and 5 mV (at 60 °C), respectively.

**Table 2:** Comparison of Tafel slopes (*b*), exchange current densities (*j*_o_), overpotential (*ƞ*) at -300 mA cm^-2^ and the difference between the overpotentials under *silent* and ultrasonic conditions (Δ*E*) for the HER on Raney-Ni in 30 wt.-% aqueous KOH solution at *T* = 25, 40 and 60 °C.

Temperature (°C)	Ultrasonic amplitude	b* (mV dec^-1^)	*j*_o_ (mA cm^-2^)	Overpotential at -300 mA cm^-2^(mV)	Δ*η* (mV)
25	0 (*silent*)	113	0.52	-308	34
	100%	105	0.77	-274	
40	0 (*silent*)	128	8.38	-203	13
	100%	135	13.07	-190	
60	0 (*silent*)	130	52.78	-140	5
	100%	144	83.73	-135	

* -^180 ≤^*^η^*^≤^ -^300 mV^

Two mechanisms can be proposed to explain the decrease in overpotential under ultrasonic conditions [9]. The first is that ultrasonically produced cavitation modifies the surface of the electrode, for instance, by changing the nature of active sites available for the adsorption of hydrogen (*H*_ad_) on the electrode surface. The erosion, caused by the implosion of high-energy cavitation bubbles, cleans and activates the electrode surface and produces nucleation sites continuously [21]. The second proposed mechanism is the degassing effect associated with micro-streaming (non-periodic motion of the fluid resulting from the propagation of the sound wave in the electrolyte) [30] together with acoustic cavitation. It is well-accepted in water electrolysis that the electrolyte adjacent to the electrode surface is supersaturated with molecular hydrogen (because of the low solubility of molecular hydrogen in aqueous solutions) leading to the so-called “bubble overpotential” or “bubble resistance” [9]. It is also known that acoustic streaming and cavitation help degas the solution immediately adjacent to the electrode, thus, decreasing and even eliminating this overpotential in some cases [9]. In our study, the observed decreased overpotentioal could be due to the elimination of gas bubbles on the surface of the electrode (second proposed mechanism) since no structural change has been observed on Raney-Ni surface after ultrasonication according to the SEM images (Figure 4, Figure 5).

Our results also show that the effect of ultrasonication decreases with increasing the electrolyte temperature. This phenomenon can be explained by the basic principle of sonochemistry in pure water. Increasing temperature decreases the polytropic index (*γ* =$\frac{c_{p}}{c_{v}}$) of gases, the *C*_p_ and *C*_v_ are the specific heats of an ideal gas at constant pressure and at constant volume, respectively. When the liquid temperature increases, it causes a less violent collapse of the cavitation bubble due to the decrease of the polytropic index. Less violent collapse leads to lower internal bubble temperatures. Lower internal bubble temperature lowers the formation of free radicals by the decomposition of water i.e. sonolysis [9]. It is also known that increasing temperature quenches the cavitation process. Therefore, increasing temperature decreases the global cavitational activity of the system leading to the decrease in the sonoelectrochemical effect [31].

The Tafel slope is a specific characteristic of the HER catalysts from which some indication about the reaction mechanism of the HER and the rate-determining step (*rds*) can be obtained. The Volmer reaction involves the electroreduction of water molecules with hydrogen adsorption as shown in Equation 10, while Heyrovsky's reaction involves electrochemical hydrogen desorption (Eq. 11). The Tafel reaction involves chemical desorption (Eq. 12) [3], [32], [33].

H_2_O + e^-^ → H_ads_ + OH^-^ Volmer (10)

H_ads_ + H_2_O + e^-^ →H_2_ + OH^-^ Heyrovsky (11)

2H_ads_ → H_2_ Tafel (12)

The HER pathway in alkaline medium follows the Volmer–Heyrovsky step or Volmer–Tafel step [25], [33], [34], [35]. The Tafel slopes for the HER in alkaline aqueous media at Ni materials and at room temperature have been reported by many researchers to be ca. 116–117 mV dec^−1^ in the low overpotential region [25]. However, lower values (e.g., < 100 mV dec^−1^), as well as higher values (e.g., > 140 or higher mV dec^−1^) of the Tafel slope, are sometimes reported for Ni materials having various forms, such as bulk Ni, porous Ni, and Raney-Ni [35], [36], [37], [38], [39], [40]. According to literature, the *rds* for the HER on Ni is usually the Volmer step [41], [42], [43]. Table 2 shows that the Tafel slopes are between 113 mV dec^-1^ and 144 mV dec^-1^ which are in good agreement with literature and the *rds* of HER on Raney-Ni in the absence and presence of ultrasound at *T* = 25, 40 and 60 °C is the Volmer reaction, suggesting that ultrasound does not change the mechanism of the HER on Raney-Ni electrodes under our conditions.

**Figure 7:**
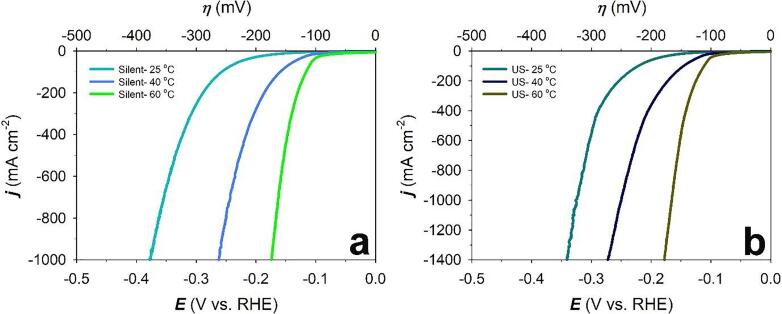
Comparison of LSV curves of HER on Raney-Ni in 30 wt.-% aqueous KOH solution in a) *silent* and b) ultrasonic (US) conditions at a scan rate of *v* = 0.3 mV s^-1^ and *T* = 25, 40 and 60 °C.

To better understand the effect of temperature on the HER on Raney-Ni, LSV curves of Raney-Ni at *T* = 25, 40 and 60 °C under *silent* and ultrasonic conditions were generated. Table 2 and Figure 7 show that the exchange current density (*j*_o_) increases and the overpotential decreases by increasing the temperature. Also, the Tafel slopes under *silent* conditions increase from 113 mV dec^-1^ (at 25 °C) to 128 mV dec^-1^ (at 40 °C) and to 130 mV dec^-1^ (at 60 °C), and the Tafel slopes under ultrasonication increases from 105 mV dec^-1^ (at 25 °C) to 135 mV dec^-1^ (at 40 °C) and to 144 mV dec^-1^ (at 60 °C). These results are in good agreement with the literature since the Tafel slope increases by increasing temperature *T* according to Equation (13) [44]:

(13) $$b=\frac{2.303RT}{\alpha F}$$

where *α* is the charge transfer coefficient, *R* is the gas constant, and *F* is the Faraday constant.

### 3.3 Effect of ultrasound and temperature on the oxygen evolution reaction

**Figure 8:**
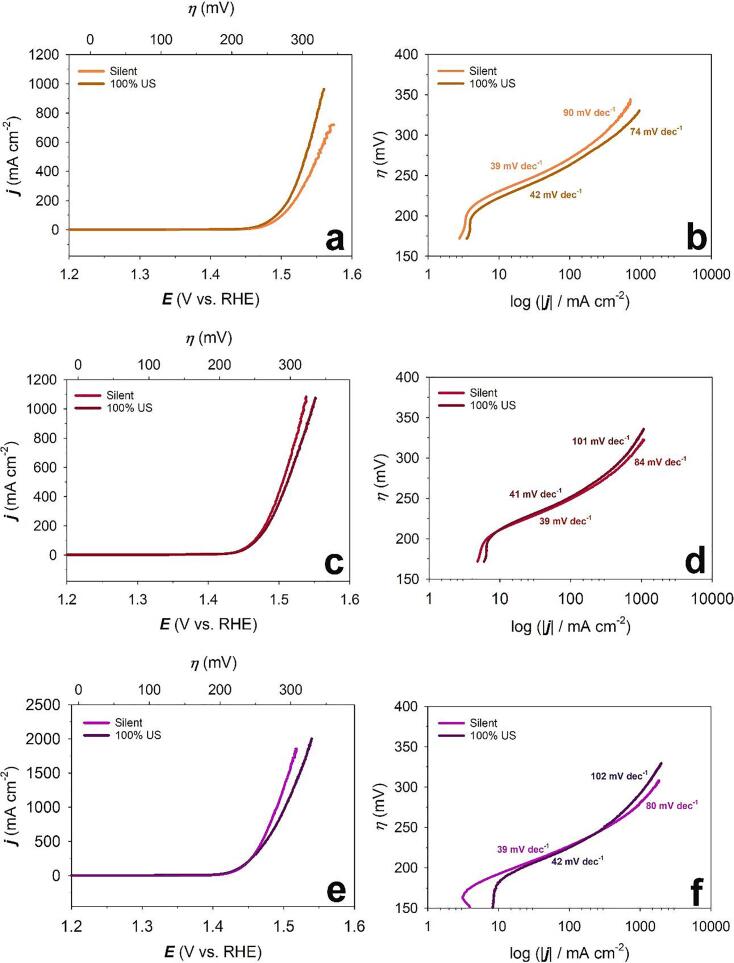
Linear sweep voltammograms (LSV) at a) 25 °C, c) 40 °C, e) 60 °C and Tafel plots of OER at b) 25 °C, d) 40 °C, f) 60 °C on Raney-Ni in 30 wt.-% aqueous KOH solution at a scan rate of *v* = 0.3 mV s^-1^ after purging with N_2_(g) for 30 min in *silent* and ultrasonic (US) conditions (*f* = 408 kHz).

Figure 8 shows the LSV curves and Tafel plots of Raney-Ni in 30 wt.-% aqueous KOH solution for the OER in the absence and presence of ultrasound at *T* = 25, 40 and 60 °C. These experiments were carried out in the applied potential region +0.20 ≤ *E*_app_ ≤ +0.80 V *vs.* Hg/HgO (+1.10 ≤ *E*_app_ ≤ +1.70 V *vs.* RHE) and at a potential scan rate of *ν* = 0.30 mV s^-1^. It is evident from the figure that ultrasound does not significantly affect the performance of Raney-Ni towards the OER compared to the HER (see before). This can be explained by the different behaviour of O_2_ and H_2_ bubble dynamics in the absence and presence of ultrasound [45].

There are three possible explanations: (i) the bubble coverage on the electrode for the OER is much higher than that for the HER in the absence of forced convection flow, (ii) the average size of H_2_ bubbles is much smaller than that of O_2_ bubbles, and (iii) the residence time of O_2_ bubbles on the electrode is much longer than that of H_2_ bubbles [45], [46]. Consequently, the O_2_ gas bubbles are much harder to remove from the electrode surface than H_2_ bubbles in our conditions, i.e., under ultrasonication at 408 kHz [45]. Li *et al.* [45] and Pollet *et al.*[20] reported that applying power ultrasound in a water electrolysis reactor improves the hydrogen production rate by 5–18% at certain current densities because the sound wave carries the hydrogen bubbles away from the electrode as soon as the hydrogen bubbles are generated and before they are able to coalesce. However, it was found that the generation of oxygen bubbles at an electrode decreases by 8% at high electrolyte concentrations under ultrasonication [45], [47].

To this day, it is still unclear why ultrasound has little effect on the oxygen evolution at an electrode, however a few researchers have attempted to explain bubble hydrodynamic under an ultrasonic field [21], [48], [49]. Under *silent* conditions, oxygen bubbles grow and coalesce with adjacent bubbles. The buoyancy force can remove the bubbles from the surface of the electrode and once the bubble reaches a certain volume, a disengagement occurs at the interphase. When the bubbles are exposed to ultrasound, the bubbles start to oscillate and then interact with the ultrasonic field, inducing the primary *Bjerknes* force [50], [51]. Then the small bubbles start moving toward the larger bubbles, influenced by the secondary *Bjerknes* force [52]. This force is induced by the difference in compressibility and density between the bubbles. After agglomeration, the bubbles coalesce due to unstable film caused by oscillation and collision [48]. It is possible that under ultrasonication, the oxygen bubble coalescence might be prevented by the oscillation dynamics on the bubbles, and since the oxygen bubbles have a larger average diameter compared to the hydrogen bubbles, it requires a higher force to overcome the surface tension at the gas-solid interphase [49]. It is also known that the gas type also affects the bubble hydrodynamics [45]. Under ultrasonication, the oxygen bubble size might not reach a critical diameter to release it from the electrode surface via buoyancy force, and thus, disengagement would not occur.

Table 3 compares Tafel slopes (*b*), exchange current densities (*j*_o_) and overpotential at +300 mA cm^-2^ (*ƞ_300_*) for the OER on Raney-Ni in 30 wt.-% aqueous KOH solution at *T* = 25, 40 and 60 °C. According to Table 3, *ƞ_300_* decreases by 13 mV (at 25 °C), and 1 mV (at 60 °C) and increases by 4 mV (at 40 °C) in presence of ultrasound. It can be observed that the Tafel slopes at low current densities are independent of temperature. Also, an increase of Tafel slope is shown at high temperatures *T* = 40 °C and 60 °C by applying ultrasound while at *T* = 25 °C the opposite behaviour can be observed. It can be suggested that ultrasound has a more pronounced effect in high current densities towards the OER.

**Table 3:** Comparison of Tafel slopes (*b*), exchange current densities (*j*_o_) and overpotential (*ƞ*) at +300 mA cm^-2^ for the OER on Raney-Ni in 30 wt.-% aqueous KOH solution at *T* = 25, 40 and 60 °C.

Temperature (°C)	Ultrasonic amplitude	*b* (mV dec^-1^) at low overpotential*	*b* (mV dec^-1^) at high overpotential**	*j*_o_ (mA cm^-2^) at low overpotential	*j*_o_ (mA cm^-2^) at high overpotential	Overpotential at +300 mA cm^-2^ (mV)
25	0 (*silent*)	39	90	1.35 × 10^-5^	0.12	302
	100%	42	74	5.51 × 10^-5^	0.038	290
40	0 (*silent*)	39	84	4.00 × 10^-5^	0.18	274
	100%	41	101	7.9 × 10^-5^	0.54	278
60	0 (*silent*)	39	80	12.00 × 10^-5^	0.30	247
	100%	42	102	45.00 × 10^-5^	1.25	248

^* 200 ≤^*^η^*^≤ 250 mV^

^** 250 ≤^*^η^*^≤ 300 mV^

The OER mechanism for catalysis can be based upon theoretical studies reported by Rossmeisl *et al.* [53]. Accordingly, oxygen evolution consists of four steps that involve three oxygen-adsorbed species (OH_ad_, O_ad_ and OOH_ad_) plus the active site (*) as intermediates in the overall process, as shown in Equations (14) to (17) [54]:

$H_{2}O+*⇌{OH}_{ad}+H^{+}+e^{-}$ (120 mV dec^-1^) (14)

${OH}_{ad}⇌O_{ad}+H^{+}+e^{-}$ (40 mV dec^-1^) (15)

(16) $$O_{ad}+H_{2}O⇌OOH_{ad}+H^{+}+e^{-}$$

with oxygen evolution finally taking place through:

(17) $$OOH_{ad}⇌*+O_{2}+H^{+}+e^{-}$$

**Figure 9:**
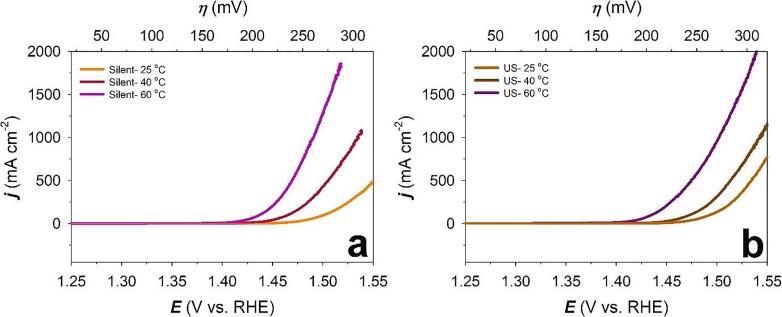
Comparison of LSV curves of OER on Raney-Ni in 30 wt.-% aqueous KOH solution in a) *silent* and b) ultrasonic (US) conditions at a scan rate of *v* = 0.3 mV s^-1^ and *T* = 25, 40 and 60 °C.

According to our results and at low current densities, deprotonation of the adsorbent (Eq. 15) is the rate-limiting step in the OER process. At higher current densities, the rate-determining step moves to the oxidative adsorption of water (Eq. 14) [54]. It must be mentioned that the production of radicals by sonolysis has to be considered but appears to be not relevant here and further studies are necessary. Exchange current density at high overpotential regions slightly increases at all the temperatures under our investigation in presence of ultrasound. By comparing the high overpotential region at different temperatures, we can see that *j*_o_ improves by increasing temperature based upon Arrhenius’ law. Also, lower overpotentials (*ƞ_300_*) can be achieved by increasing temperature (Figure 9).

### 3.4 Electrochemical impedance spectroscopy (EIS) at different temperatures

**Figure 10:**
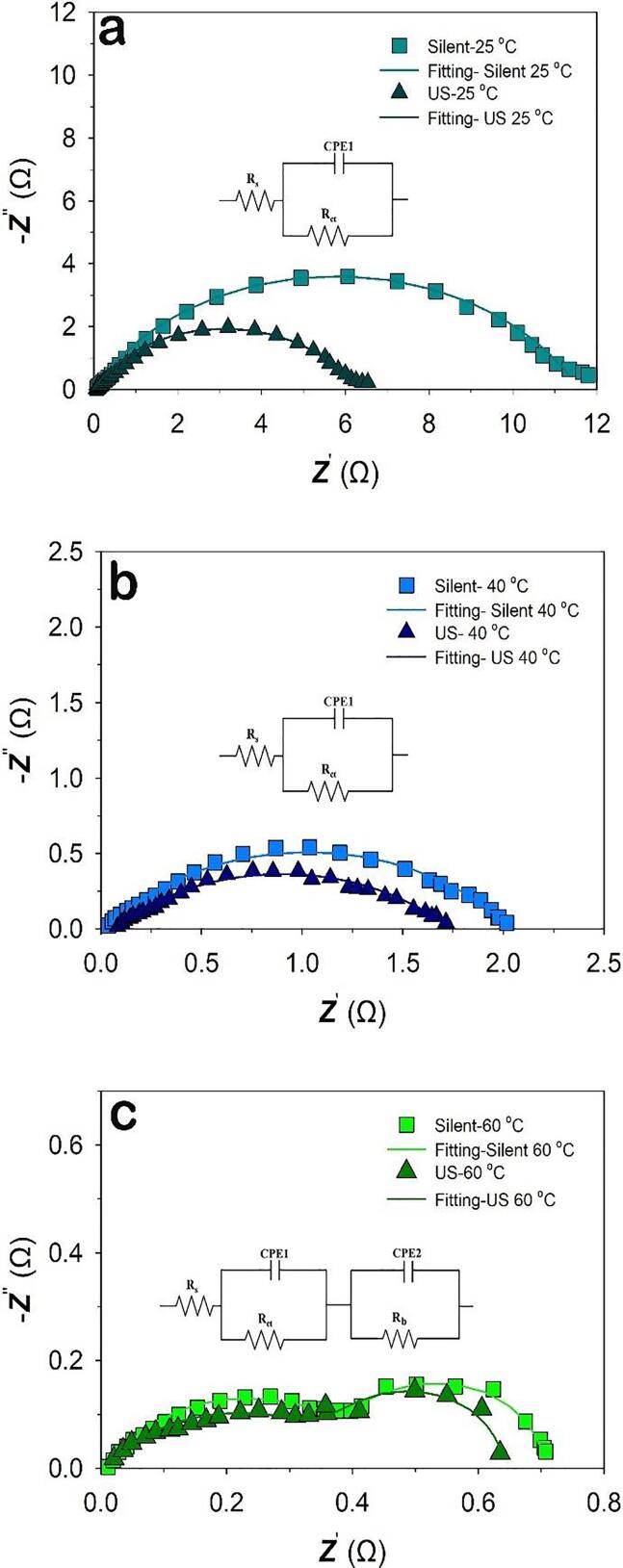
Nyquist plots of Raney-Ni in 30 wt.-% aqueous KOH under *silent* and ultrasonic (US) conditions at a) 25 °C, b) 40 °C, c) 60 °C, at the potential *E* = -200 mV *vs.* RHE; Inset figures show the equivalent circuit used to fit the impedance data.

Figure 10 shows the Nyquist representation of the impedance data of Raney-Ni under *silent* and ultrasonic conditions, at *T* = 25 °C, 40 °C and 60 °C and *E* = -200 mV *vs.* RHE. In both conditions at *T* = 25 °C and 40 °C, a depressed semi-circle can be seen which can be attributed to the porosity or surface roughness of the electrode [55], [56], [57]. Accordingly, the data at *T* = 25 and 40 °C were approximated by the modified Randles circuit shown in Figure 10, whereas the capacitance is replaced by a constant phase element. Note, for α = 1 the CPE reflects an ideal capacitance. However, at *T* = 60 °C the impedance consists of two partly overlapping and depressed semi-circles. The fitted electrical circuit is comprised of two RC parallel combinations in series with a resistor. *R*_s_ correlates with the cell Ohmic resistance (electrodes and current collectors). *R*_ct_ represents the charge transfer resistance and may also include other contributions such as the adsorption of intermediates. CPE1 is a constant phase element that represents the capacitive charging of a rough electrode. The equivalent circuit has an extra R-CPE combination, where CPE2 and *R*_b_ are suggested to represent the formation of bubbles and mass transport at the electrode−electrolyte interface [58]. The parameters obtained from the EIS measurement are shown in Table 4. The capacitance (*C*) can be calculated by using Equation (18) [55], [57].

(18) $$C={\left[Q_{1}{\left(\frac{1}{R_{s}}+\frac{1}{R_{\mathrm{ct}}+R_{b}}\right)}^{\left(a-1\right)}\right]}^{\frac{1}{a}}$$

**Table 4:** Parameters obtained from the EIS measurements at *E* = -200 mV *vs.* RHE.

Temperature (°C)	Ultrasonic amplitude	*R*_s_ (Ω)	*R*_ct_ (Ω)	*R*_b_ (Ω)	a	*Q*_1_ (mF s^(a-1)^)	*C* (mF cm^-2^)
25	0 (*silent*)	2.55	11.59	-	0.70	5.27	8.98
	100 %	2.07	6.31	-	0.70	8.17	14.40
40	0 (*silent*)	1.74	2.01	-	0.60	25	25.88
	100%	1.78	1.67	-	0.52	48	41.18
60	0 (*silent*)	1.16	0.49	0.29	0.62	49	47.06
	100%	1.04	0.47	0.24	0.58	43	36.47

where *Q_1_* is the parameter of the constant phase element (CPE1), α is related to the phase angle of the frequency response, *R*_s_ is the Ohmic resistance, *R*_ct_ is the charge transfer resistance and *R*_b_ is the resistance due to the formation of bubbles. The CPE (constant phase element) instead of a real capacitance was used because a depressed semi-circle is observed. Furthermore, the increase of the parameter of the constant phase element, *Q* is mainly due to the enhancement of the active surface area. Table 4 presents the temperature dependence of the Ohmic resistance and charge transfer resistance*.* For the three temperatures i.e., 25, 40 and 60 °C, the recorded Ohmic and charge transfer resistance exhibited significant reduction by increasing the temperature. In addition, the capacitance increases by elevating the temperature which indicates a higher active surface area by rising the temperature. The above could be explained in terms of extended access to the catalytic surface within the electrode structure at elevated temperatures. In addition, higher temperatures should considerably facilitate hydrogen bubble removal [59].

As stated earlier, the effect of ultrasonication decreases with increasing the electrolyte temperature. It was found that at 25 °C and under ultrasonication, the charge transfer resistance was almost half compared to *silent* conditions. While at 60 °C, a slight decrease of *R*_ct_ was observed. In addition, higher capacitance values at *T* = 25 °C and 40 °C were observed in presence of ultrasound. The EIS results underpin our assumption that ultrasonication has mainly an impact on the gas bubble release, i.e., it can easily remove the gas bubbles from the electrode surface and bulk electrolyte to reduce the bubble surface coverage of the electrodes which in turn increase the active sites for the further reaction [21]. The Tafel as well as EIS results show that the electrode global HER activity increases with temperature (exemplarily outlined by the *R*_ct_, *C*, and *j*_o_ values). Since HER is already greatly improved at high temperatures, adding ultrasonication does not greatly improve the HER when the electrolyte temperature is increased. Moreover, ultrasonication cannot overcompensate the reduced activity by lowering the temperature.

**Figure 11:**
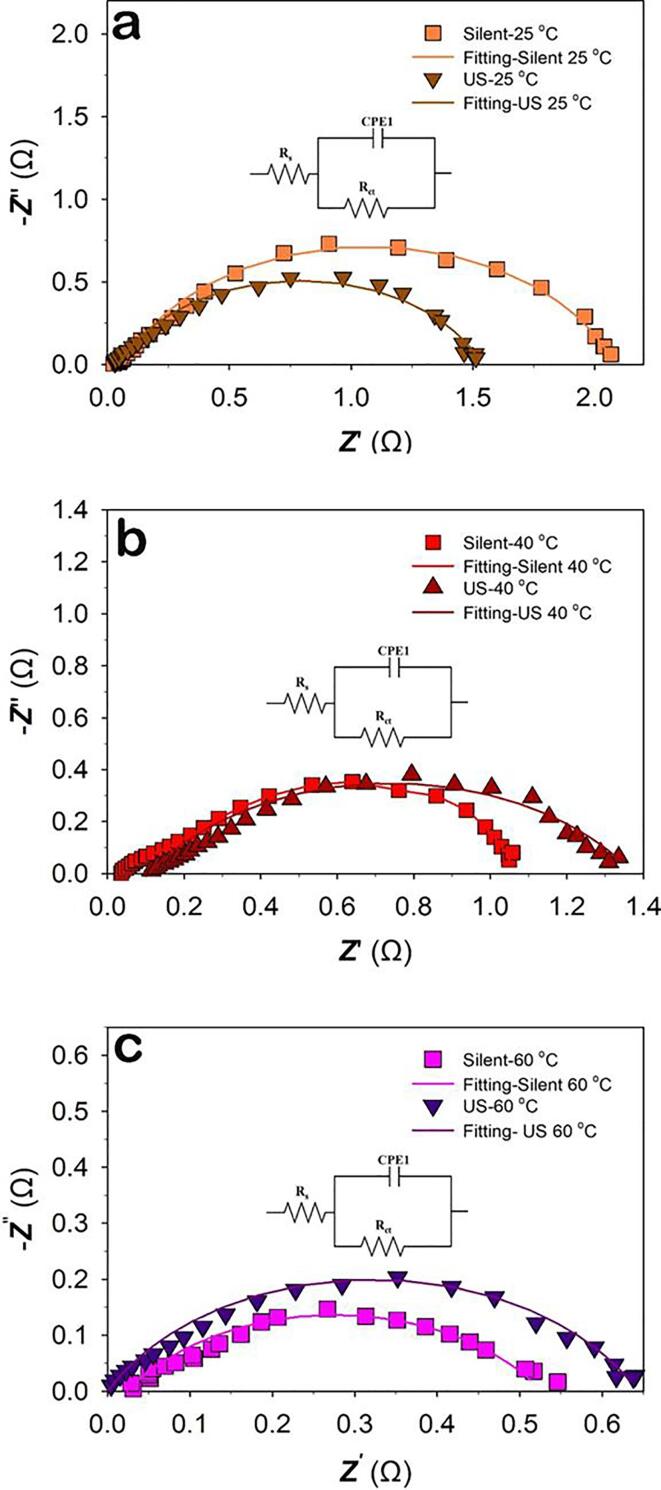
Nyquist plots of Raney-Ni in 30 wt.-% aqueous KOH under *silent* and ultrasonic (US) conditions at a) 25 °C, b) 40 °C , c) 60 °C, at the applied potential *E* = +1,550 mV vs. RHE; Inset figures show the equivalent circuit used to fit the impedance data.

Figure 11 shows the Nyquist representation of the impedance data of Raney-Ni under *silent* and ultrasonic conditions at *T* = 25, 40 and 60 °C at *E* = +1,550 mV *vs.* RHE in the OER region. Figure 11 shows depressed semi-circles which can be fitted to a modified Randles circuit as described above. The values for each circuit element/parameter are given in Table 5. Figure 11 and Table 5 show that under ultrasonic conditions the charge transfer resistance slightly decreases at 25 °C while at 40 and 60 °C, the charge transfer resistance increases to a slight extent. The EIS data are in good agreement with the Tafel results and illustrate that ultrasonication does not influence the mass transfer of oxygen bubbles, especially at high temperatures. In short, both the EIS and Tafel data indicate that ultrasound has a different effect on the HER and OER. While it has significant influence on the hydrogen bubble release from the electrode surface, it does not affect the O_2_ bubble surface coverage at the ultrasonic frequency used (408 kHz). This may be due to the fact that the dynamic behaviour of O_2_ gas bubbles for alkaline water electrolysis is different from that of H_2_ bubbles [21], [46] and the ultrasound effect is directly related to gas bubbles size in electrochemical reactions [21], [49].

**Table 5:** Parameters obtained from the EIS measurements at *E* = +1,550 mV *vs.* RHE in the OER region.

Temperature (°C)	Ultrasonic amplitude	*R*_s_ (Ω)	*R*_ct_ (Ω)	a	*Q*_1_ (mF s^(a-1)^)	*C* (mF cm^-2^)
25	0 (*silent*)	2.126	2.015	0.78	28	117.7
	100 %	1.757	1.494	0.76	35	129.4
40	0 (*silent*)	1.878	0.97	0.78	82	423.5
	100%	1.725	1.27	0.64	73	494.1
60	0 (*silent*)	1.178	0.50	0.63	135	282.1
	100%	0.939	0.64	0.71	64	200.0

## 4 Conclusions

The electrochemical kinetics and mechanism of Raney-Ni towards the HER and the OER under *silent* and ultrasonic (408 kHz) conditions have been investigated in 30 wt.- % aqueous KOH solution at different temperatures (*T* = 25, 40 and 60 °C). It was observed that there is a significant difference between the effect of ultrasonication on the HER and the OER. Ultrasonication significantly shifts the overpotential at -300 mA cm^-2^ (*ƞ_300_*) of HER by +34 mV at 25 °C due chiefly to the effective bubble removal while it does not influence the OER overpotential. This may be attributed to the direct dependence of the ultrasonic effect on the difference of O_2_ and H_2_ gas bubble sizes and dynamic behaviours. It was also shown that the ultrasonic effect on the HER depends upon temperature and ultrasonication does not play a remarkable role at high temperatures since at these temperatures, the HER is already very efficient. In addition, increasing the electrolyte temperature decreases the global cavitational activity of the system leading to a decrease in the sonoelectrochemical effect. Moreover, ultrasonication cannot overcompensate the decreasing HER activity by lowering the temperature. This study has highlighted some improvements that can be achieved using power ultrasound and the results obtained were indicative of some benefits and improvements to water electrolysis. Also, for the first time, the Tafel plots and mechanism of HER and OER on Raney-Ni under ultrasonication at different temperatures have been reported.

These preliminary findings might be helpful for experimentalists that intend to use power ultrasound in energy storage and energy conversion for hydrogen production. The performance of the sonoelectrochemical technique can be improved by the optimization of various operating conditions and parameters as follows [10]: an ultrasonic probe-type emitter is preferable for producing high-intensity bubbles and free radical formation since ultrasonic frequencies are mostly in the range of 20–100 kHz. The rate of electrochemical reaction rate mostly increases by increasing the acoustic power and intensity. Lower ultrasonic frequency is preferred over higher frequencies to improve mass transfer at the electrode. Electrode materials selection is important for efficient sonoelectrochemical processes. Finally, optimization of different experimental parameters, such as experimental design, ultrasonic frequency, acoustic power, ultrasonic transducer–electrode distance, irradiation time, electrode materials, electrode potentials, temperature, pH, conductivity, and electrolyte compositions are recommended for efficient sonoelectrochemical processes.

## Declaration of Competing Interest

The authors declare that they have no known competing financial interests or personal relationships that could have appeared to influence the work reported in this paper.
